# Expression of Terpenoid Biosynthetic Genes and Accumulation of Chemical Constituents in *Valeriana fauriei*

**DOI:** 10.3390/molecules21060691

**Published:** 2016-05-27

**Authors:** Yun Ji Park, Mariadhas Valan Arasu, Naif Abdullah Al-Dhabi, Soon Sung Lim, Yeon Bok Kim, Sang Won Lee, Sang Un Park

**Affiliations:** 1 Department of Crop Science, Chungnam National University, 99 Daehak-ro, Yuseong-gu, Daejeon 34134, Korea; yunji0825@hanmail.net; 2 Department of Botany and Microbiology, Addiriyah Chair for Environmental Studies, College of Science, King Saud University, P. O. Box 2455, Riyadh 11451, Saudi Arabia; mvalanarasu@gmail.com (M.V.A.); naldhabi@ksu.edu.sa (N.A.A.-D.); 3 Department of Food and Nutrition and Institute of Natural Medicine, Hallym University, Chuncheon 200-702, Korea; limss@hallym.ac.kr; 4 Department of Herbal Crop Research, National Institute of Horticultural and Herbal Science (NIHHS), Rural Development Administration (RDA), Bisanro 92, Eumseong, Chungbuk 369-873, Korea; yeondarabok@korea.kr (Y.B.K.); swlee1004@korea.kr (S.W.L.)

**Keywords:** gene expression, terpenoid, valerenic acid, *Valeriana fauriei*, volatile compounds

## Abstract

*Valeriana fauriei* (*V. fauriei*), which emits a characteristic and unpleasant odor, is important in traditional medicine. In this study, the expression of terpenoid biosynthetic genes was investigated in different organs that were also screened for volatile compounds including valerenic acid and its derivatives. Specific expression patterns from different parts of *V. fauriei* were observed using quantitative real-time PCR (qRT-PCR). The highest transcript levels of biosynthetic genes involved in mevalonic acid (MVA) and methylerythritol phosphate (MEP) production were found in the stem. Although the amounts of volatile compounds were varied by organ, most of the volatile terpenoids were accumulated in the root. Gas chromatography mass spectrometry (GC-MS) analysis identified 128 volatile compounds, which represented 65.33% to 95.66% of total volatiles. Certain compounds were only found in specific organs. For example, isovalerenic acid and valerenic acid and its derivatives were restricted to the root. Organs with high transcript levels did not necessarily have high levels of the corresponding chemical constituents. According to these results, we hypothesize that translocation may occur between different organs in *V. fauriei*.

## 1. Introduction

Plants have complex mixtures of volatile lipophilic compounds with low molecular weight and high vapor pressure, derived from both primary and secondary metabolisms [1]. More than 1700 volatile compounds have been characterized from 90 plant families [2]. Plants release these compounds for general or specialized functions in both floral and vegetative tissues [3]. These compounds protect against herbivores, pathogens, and parasites, attract pollinators and seed dispersers, and provide plant–plant signaling [4]. Additionally, volatile compounds emitted from plants can seal wounds [5]. For millennia, humans have used floral scents from many aromatic plants, intended to attract pollinators, as sources of flavorings, preservatives, and herbal remedies [6]. Researches have documented antimicrobial, anti-inflammatory, bronchodilatory, expectorant, anticonvulsant, cholagogic, analgesic, and spasmolytic effects of volatile compounds [7]. Volatile compounds determine a plant’s chemotype and can influence its ecological relevance by shaping interactions with pollinators and herbivores [8]. Diverse volatile compounds are synthesized by different biochemical pathways in plants. There are lipoxygenase pathways including oxylipins, green leaf volatiles, isoprene and other terpenoids, some carotenoid derivatives, indoles, and phenolics, such as methyl salicylate [9].

**Figure 1:**
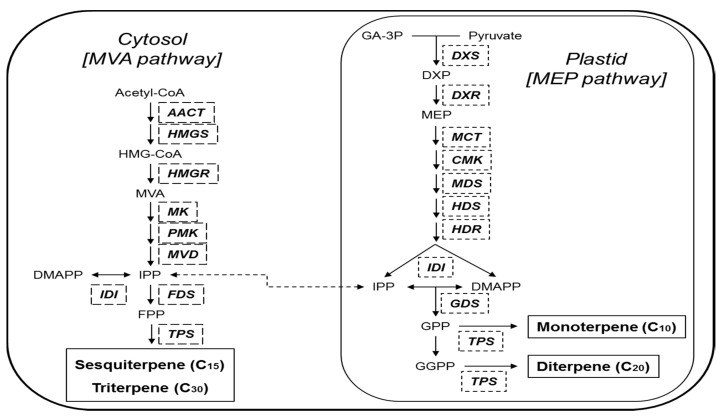
Volatile terpenoid biosynthetic pathways in plants. *AACT*, Acetoacetyl-CoA thiolase; *CMK*, 4-(cytidine 5′-diphosphate)-2-*C*-methyl-d-erythritol kinase; *DMAPP*, dimethylallyl diphosphate; DXP, 1-deoxy-d-xylulose 5-phosphate; *DXR*, DXP reductoisomerase; *DXS*, DXP synthase; *FDS*, farnesyl diphosphate synthase; FPP, farnesyl diphosphate; GA-3P, glyceraldehyde-3-phosphate; *GDS*, geranyl diphosphate synthase; GGPP, geranylgeranyl diphosphate; GPP, geranyl diphosphate; *HDR*, *(E)*-4-hydroxy-3-methylbut 2-enyl diphosphate reductase; *HDS*, *(E)*-4-hydroxy-3-methylbut-2-enyl diphosphate synthase; HMG-CoA, 3-hydroxy-3-methylglutaryl-CoA; *HMGR*, HMG-CoA reductase; *HMGS*, HMG-CoA synthase; *IDI*, isopentenyl diphosphate isomerase; IPP, isopentenyl diphosphate; *MCT*, 2-*C*-methyl-d-erythritol 4-phosphate cytidylyltransferase; *MDS*, 2-*C*-methyl-d-erythritol 2,4-cyclodiphosphate synthase; MEP, 2-*C*-methyl-D-erythritol 4-phosphate; *MK*, mevalonate kinase; MVA, mevalonate; *MVD*, mevalonate diphosphate decarboxylase; *PMK*, phosphomevalonate kinase; *TPS*, terpene synthases.

Terpenoids, which constitute the largest class of volatiles from plants, are synthesized from isoprenoid pathways (Figure 1) [1]. There are three basic phases of volatile terpenoid biosynthesis [10]. First, C_5_-isoprene units are formed from isopentenyl pyrophosphate (IPP) and its isomer dimethylallyl pyrophosphate (DMAPP) via two compartmentally separated pathways, the mevalonic acid (MVA) pathway in the cytoplasm and the methylerythritol phosphate (MEP) pathway in the chloroplast. These precursors are then catalyzed by short-chain prenyltransferase to form prenyl diphosphates, including geranyl diphosphate (GPP), farnesyl diphosphate (FPP), and geranylgeranyl pyrophosphate (GGPP) [11,12]. Finally, many terpene synthases (TPS) synthesize prenyl diphosphates and build primary representatives of each type of terpenoid skeleton. Many volatile terpenoids are directly catalyzed by TPS, but others are modified by oxidation, dehydrogenation, acylation, and other types of reactions [10]. The MVA pathway is thought to provide three C_5_ units for volatile sesquiterpenes (C_15_), while the MEP pathway gives rise to volatile hemiterpenes (C_5_), monoterpenes (C_10_), and diterpenes (C_20_) [13]. However, metabolic cross talk has been demonstrated, regulated by exchange of isoprenes like IPP between cytosolic and chloroplastic pathways [14].

The genus *Valeriana* L. belongs to Caprifoliaceae (honeysuckle family), and includes approximately 250 perennial herbaceous species with malodorous root stalks [15], and grows in temperate areas around the world [16]. These species have been used as mild sedatives and tranquilizers in traditional medicine in various cultures since ancient times [17]. The rhizomes and roots are thought to induce sedation, promote sleep, and relieve depression and anxiety. Species of *Valeriana* are also used in the food, beverage, and cosmetic industries because of their unique flavor [18]. Many studies have demonstrated that extracts from *Valeriana* plants include terpenoids, iridoids, flavonoids, and alkaloids [16]. Valerenic acid and its derivatives accumulate in the roots and rhizomes in significant quantities [19]. Chemical constituents of *Valeriana* vary by species and with seasonal variation and ecological factors [16,17].

We profiled overall volatile compounds, including valerenic acid and its derivatives, from different tissues of *Valeriana fauriei* (*V. fauriei*) grown in South Korea. In addition, we characterized the expression patterns of terpenoid biosynthetic genes and compared them with patterns of volatile compound accumulation.

## 2. Results

### 2.1. Transcript Levels of Terpenoid Biosynthetic Genes

**Figure 2:**
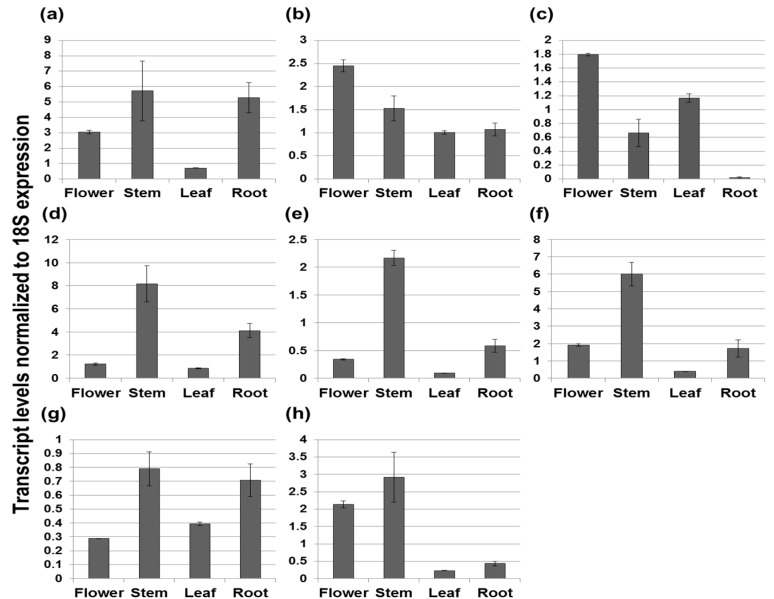
Transcript levels of MVA biosynthetic genes in different organs of *V. fauriei* (*Vf*). Genes encode the following enzymes: (**a**) *VfAACT*, acetoacetyl-CoA thiolase; (**b**) *VfHMGS*, 3-hydroxy-3-methylglutaryl-CoA synthase; (**c**) *VfHMGR*, 3-hydroxy-3-methylglutaryl-CoA reductase; (**d**) *VfMK*, mevalonate kinase; (**e**) *VfPMK*, phosphomevalonate kinase; (**f**) *VfMVD*, mevalonate diphosphate decarboxylase; (**g**) *VfFDS*, farnesyl diphosphate synthase; and (**h**) *VfIDI*, isopentenyl diphosphate isomerase.

**Figure 3:**
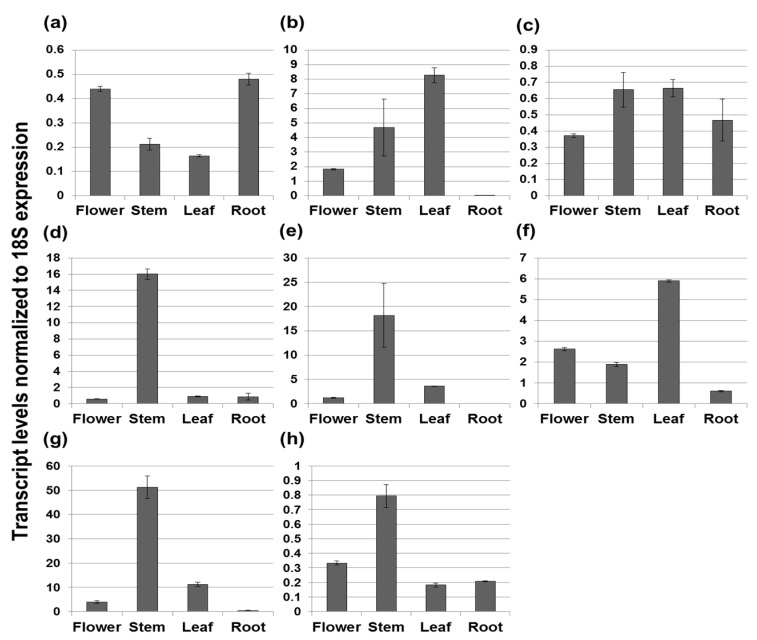
Transcript levels of MEP biosynthetic genes in different organs from *V. fauriei* (*Vf*). Genes encode the following enzymes: (**a**) *VfDXS*, 1-deoxy-d-xylulose 5-phosphate synthase; (**b**) *VfDXR*, 1-deoxy-d-xylulose 5-phosphate reductoisomerase; (**c**) *VfMCT*, 2-*C*-methyl-d-erythritol 4-phosphate cytidylyltransferase; (**d**) *VfCMK*, 4-(cytidine 5’-diphosphate)-2-*C*-methyl-d-erythritol kinase; (**e**) *VfMDS*, 2-*C*-methyl-d-erythritol 2,4-cyclodiphosphate synthase; (**f**) *VfHDS*, (*E*)-4-hydroxy-3-methylbut-2-enyl diphosphate synthase; (**g**) *VfHDR*, (*E*)-4-hydroxy-3-methylbut 2-enyl diphosphate reductase; and (**h**) *VfGDS*, geranyl diphosphate synthase.

We determined differential organ-specific expression patterns of terpenoid biosynthetic genes in *V. fauriei* using qRT-PCR (quantitative real time polymerase chain reaction). The results are shown in Figure 2 and Figure 3. The height of each bar and the error bar indicate the mean and standard deviation based on three independent measurements. The Y-axis represents transcript levels normalized to *18S* expression. Most genes related to the MVA pathway showed the highest expression levels in the stem, except for *VfHMGS* and *VfHMGR*. The expression patterns of *VfAACT*, *VfMK*, *VfPMK*, *VfMVD*, and *VfFDS* were fairly similar, with the highest levels occurring in the stem, followed by the root and the flower or the leaf. Interestingly, in roots, *VfAACT* and *VfFDS*, which are involved in the first and last steps of the MVA pathway, were expressed at similar levels to the stem. Among MVA biosynthetic genes, the expression of *VfAACT*, *VfMK* and *VfMVD* was high relative to *18S*, while expression of *VfFDS* was the lowest relative to *18S*. *VfIDI*, which produces the enzyme that catalyzes the isomerization of IPP to DMAPP in both the MVA and MEP pathways, was expressed most abundantly in the stem, followed by the flower, root, and leaf. According to analyses of MEP biosynthetic gene expression levels, most genes, including *VfCMK*, *VfMDS*, *VfHDR*, and *VfGDS*, had the highest expression levels in the stem, whereas *VfDXR* and *VfHDS* were highly expressed in the leaf. *VfMCT* had similar expression levels in all organs of *V. fauriei*. The lowest expression levels of *VfDXR*, *VfMDS*, *VfHDS*, and *VfHDR* were displayed in the root. In contrast, *VfDXS* transcripts, which are involved in the start of the MEP biosynthetic pathway, had their highest level in the root. Expression levels of terpenoid backbone biosynthetic genes, which lead to IPP and DMAPP production, were primarily high in stem of *V. fauriei*.

### 2.2. Analysis of Volatile Constituents

In total, 128 volatile compounds were separated and identified based on comparison of the mass spectra with the National Institute of Standards and Technology (NIST, Gaithersburg, MD, USA) atomic spectra database, Wiley Registry of Mass Spectral Data, and the related literature (Table 1) [20]. The total yield of the volatile compounds was indicated by the highest amount in the root (95.66%), followed by stem (84.05%), leaf (74.91%), and flower (65.33%). We identified 18 monoterpenes, 34 sesquiterpenes, and 80 other volatile compounds from *V. fauriei*. Monoterpenes, namely borneol (**11**, 22.96%), bornyl acetate (**13**, 19.92%), and camphene (**1**, 3.33%) were found in high amounts in the roots. Interestingly, β-terpinene (**3**), myrcene (**4**), *p*-cymene (**5**), and borneol (**11**) were detected only in the root. The leaf extract was characterized by a high content of *endo*-borneol (**10**, 11.48%) and γ-terpinene (**7**, 3.23%). Sesquiterpenes accumulated primarily in the stem (21.55%) and roots (20.66%). The stem contained the highest amount of the sesquiterpene caryophyllene oxide (**34**, 17.45%). Seven sesquiterpenes (**20**–**22**, **24**–**26**, and **50**) were root-specific, while four others (**33**, **37**, **39**, and **51**) were flower-specific. Isovalerenic acid (**53**, 3.33%), which produces the strong smell of *Valeriana* species, was found only in the root of *V. fauriei*.

### 2.3. Amounts of Valerenic Acid and Its Derivatives

Valerenic acid and its derivatives, acetoxyvalerenic acid and hydroxyvalerenic acid, were extracted from different organs (flower, stem, leaf, and root) of *V. fauriei* and analyzed with HPLC. Valerenic acid and its derivatives were detected only in roots (Table 2). However, flowers, stems, and leaves have not shown detectable levels of the three compounds. The root extract had an average of 69.450 μg of valerenic acid per g of dry weight. Acetoxyvalerenic acid (32.234 μg/g dry weight) also had a high accumulation in the root. However, hydroxyvalerenic acid was not detected in any organ.

## 3. Discussion

There is considerable interest in chemical composition variation in *Valeriana* owing to the numerous beneficial properties attributed to consumption of the genus. Previously, the genes involved in volatile terpenoid biosynthesis had not been investigated in different organs of *V. fauriei*. Our study documents the compositional diversity and amount of volatile compounds, including valerenic acid and its derivatives, and quantifies the expression of genes involved in terpenoid biosynthetic pathways, in different organs of *V. fauriei*.

Roots had high amounts of volatile compounds but low transcript levels. Evidently, expression levels of terpenoid biosynthetic genes did not correspond with the storage of volatile constituents, indicating that products synthesized in the stem or other organs may be translocated into the root. Earlier experimental studies have found similar results. Plants often transport natural products from a synthesis site to an accumulation site [21]. For example, compounds were synthesized in the stem and leaf, then transported to the root of *Astragalus membranaceus* Moench [22]. In addition, Lykkesfeldt and Moller [23] have reported the synthesis and translocation of glucosinolates from leaves to seeds during development in *Tropaeolum majus* L. Moreover, nicotine and caffeine, which are primarily produced roots, are transferred to leaves in *Arabidopsis thaliana* (L.) Heynh. [24]. Translocation of secondary metabolites from source cells to neighboring cells, or even further to other tissues usually occurs in plants. Volatile terpenes are emitted by the transcript levels of biosynthetic enzymes, and transporter for their emission is not required. However, the strong evidence which demonstrates to deny the presence of broad-specificity transporters for the emission of volatiles has not yet been identified. In addition, the mechanism for the long-distance translocation of volatiles has not been investigated in plants [25]. Therefore, we should carry out more experiments such as feeding of radio labels to *V. fauriei* tissues to clarify the transport and accumulation of volatile compounds in further study.

Various taxa of *Valeriana*, including *V. officinalis* L., *V. jatamansi* Jones, *V. officinalis* var. *latifolia* Miq., *V. amurensis* P. Smirn. ex Kom., *V. fauriei*, and *V. alternifolia* Bunge var. *stolonifera* A.I. Baranov & Skvortsov, have been the focus of scientific studies [18,26,27]. Several studies have included experiments to isolate and identify chemical constituents including sesquiterpenoids and iridoid glycosides from *V. fauriei* [26,27]. In our study, we identified 128 volatile compounds in *V. fauriei* by GC/MS. Although most volatile compounds had high concentrations in the roots, each organ had several distinctive compounds that served as biochemical markers. For example, isovalerenic acid, which causes the malodorous characteristic, was accumulated solely in the root of *V. fauriei*. In addition, we found that borneol and bornyl acetate were the most abundant monoterpenes in the roots of *V. fauriei*, in agreement with the results from previous studies. Wang *et al.* [18] documented that *V. officinalis* var. *latifolia* contained the highest amounts of bornyl acetate (23.93%) in the roots. To date, more than 100 members of TPSs have been identified from various plant species [13]. The action of these enzymatic family lead to structurally diverse cyclic and acyclic monoterpenes and sesquiterpenes [28]. Specific monoterpene synthase catalyze from GPP to various skeletons which are the precursors such as olefins, alcohols and diphosphate esters for the biosynthesis of monoterpenes [29]. Also, the enzymatic cyclization in several plants including *Salvia*, *Mentha*, *Tanacetum*, *Foeniculum*, *Pinus*, and *Citrus* species has been reported [30]. Cyclization of linalyl diphosphate derived from GPP occurs to form the α-terpinyl cation, which is the universal monocyclic intermediate. Then, bornane family such as borneol, bornyl acetate, and camphor is converted by hydrolysis, oxidation, and rearrangement of carbocylic skeleton [31]. It has been investigated that bornyl diphosphate synthase acts on production of bornane-type monoterpenes in higher plants [32]. The characteristic compound valerenic acid, along with its derivatives and iridoids, are primarily accumulated in the roots and rhizomes of *Valeriana* species [20].Similarly, we found that high amounts of valerenic acid and hydroxyvalerenic acid were detected only in the roots of *V. fauriei*. Navarrete *et al.* [33] performed quantitative analyses of valerenic acids (valerenic, hydroxyvalerenic, and acetoxyvalerenic) in *Valeriana* species including *V. officinalis*, *V. edulis* Nutt. ex Torr. & A. Gray, *V. sitchensis* Bong., and *V. jatamansi*, but only *V. officinalis* and *V. sitchensis* accumulated these compounds.

In summary, we analyzed organ specificity in general-common MVA and MEP biosynthesis by measuring transcript levels of related biosynthetic genes. We also measured the accumulation of volatile compounds, and the presence of valerenic acid and its derivatives, in specific organs. We separated and identified 128 volatile compounds in *V. fauriei* cultivated in South Korea. Volatile compounds were detected in all tested organs, but most of the volatile compounds were stored in the root. In contrast, high levels of gene expression predominantly occurred in the stem. Therefore, terpenoids appear to be translocated after biosynthesis and are stored in specific organs.

## 4. Materials and Methods

### 4.1. Plant Materials and Growth Conditions

**Figure 4:**
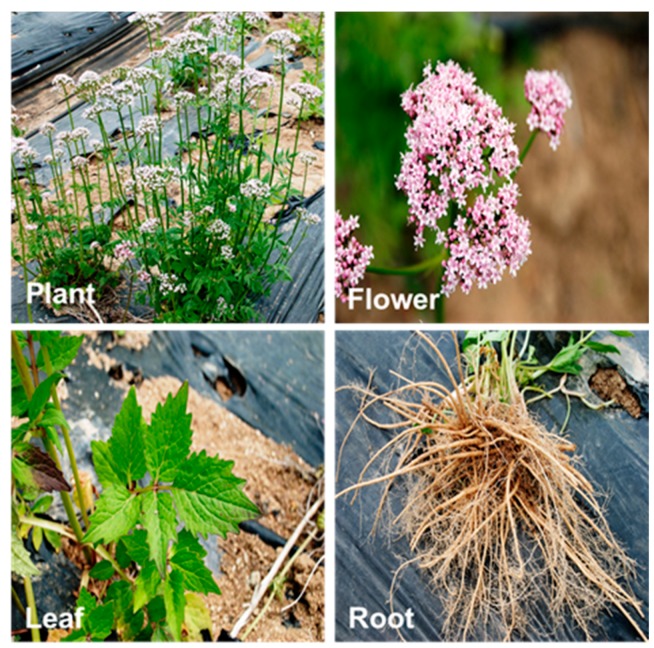
Photographs of two-year-old *V. fauriei* grown in Pyeongchang, Gangwon-do, South Korea.

*V. fauriei* plants were grown at the experimental station, National Institute of Horticultural and Herbal Science (NIHHS), Rural Development Administration (RDA), Pyeongchang, Gangwon-do, South Korea. The plants were grown outdoors in a field from 2013 to 2014. Leaves, stems, flowers, and roots were collected in July 2014 (Figure 4) and then immediately frozen in liquid nitrogen. Different organs from three individual plants were harvested under the same experimental settings. The materials were stored at −80 °C until they were used.

### 4.2. Total RNA Extraction and cDNA Preparation

Total RNA was isolated from each organ using a Total RNA Mini Kit (Geneaid, New Taipei City, Taiwan) following the manufacturer’s instructions using three biological replicates. DNase I (Qiagen, Hilden, North Rhine-Westphalia, Germany) was used to remove genomic DNA. RNA concentration and purity were confirmed with agarose gel electrophoresis and a NanoVue Plus Spectrophotometer (GE Healthcare Bio-Science Corp., Piscataway, NJ, USA). cDNA was synthesized from 1 μg of total RNA using the ReverTra Ace-α Kit (Toyobo, Osaka, Japan).

### 4.3. qRT-PCR Analysis

All gene specific primers used were designed as previously described by Park *et al* [34] (Table A1). qRT-PCR was performed with a BIO-RAD CFX96 Real-time PCR system (Bio-Rad Laboratories, Hercules, CA, USA) with the 2X Real-Time PCR smart mix (Solgent Co., Ltd. Daejeon, Korea) under the following conditions: initial denaturation at 95 °C for 15 min, 39 cycles of denaturation for 20 s at 95 °C and annealing for 20 s at 72 °C, with a final extension at 72 °C for 10 min. Melting curves were analyzed to verify reaction specificity. Means and standard deviations were calculated from three independent biological replicates for each sample and compared by organ.

### 4.4. GC and GC-MS

Samples (10 g) of the fresh material (leaves, stems, flowers, and roots) were weighed and placed in vials with 25 mL of headspace. A fused-silica fiber covered with a 75-μm-thick layer of carboxen/polydimethylsiloxane (CAR/PDMS) was used to absorb volatile compounds. It was exposed in the headspace in the vials at 25 °C for 20 min, then removed from vials and introduced directly into the GC injector where thermal desorption of the analysis was performed at 250 °C for 3 min.

GC analysis was carried out with an Agilent 6890N GC mainframe fitted with an HP-5 fused-silica capillary column (30 m × 0.32 mm i.d., 0.25 μm film thickness; Agilent, Santa Clara, CA, USA) and a flame ionization detector (FID). The injector and detector temperatures for each analysis were 250 °C and 280 °C, respectively. The carrier gas was nitrogen, with a flow rate of 1.0 mL/min. The column temperature was maintained at 50 °C for 5 min and then programmed as follows: increase from 50 °C to 260 °C at a rate of 3 °C·min^−1^, increase from 260 °C to 280 °C at a rate of 10 °C·min^−1^, and hold at 280 °C for 5 min.

GC-MS analysis was performed on a Polaris Q GC/MS (Thermo Finnigan, Waltham, MA, USA) coupled with an HP-5 fused-silica capillary column (30 m × 0.32 mm i.d., film thickness 0.25 μm; Agilent). Helium was used as the carrier gas at a flow rate of 1.0 mL/min. The mass spectra were obtained with an ionization voltage of 70 eV, trap current of 250 μA, and ion source temperature of 200 °C. The oven temperature program was the same as described previously, and injections were made in splitless mode. Volatile compounds were separated and identified based on comparison of the mass spectra with the National Institute of Standards and Technology (NIST) atomic spectra database, Wiley Registry of Mass Spectral Data, and the related literature. Total ion current chromatograms were recorded in a mass range of 40–400 amu. The analyses were performed using three biological replicates.

### 4.5. Measurement of Valerenic Acid and Derivatives

All samples were lyophilized at −80 °C for 72 h. Dried samples were ground into a fine powder using a mortar and pestle. One g of powdered sample was extracted with 10 mL of 90% (*v*/*v*) methanol at room temperature for 30 min and then centrifuged at 19354 rcf for 10 min. The supernatant was transferred to a new tube. Centrifugation and supernatant transfer were repeated two more times. The final extract was adjusted to 1 mL through evaporation using a LABOROTA 4000 rotary evaporator (Heidolph Instruments GmbH, Schwabach, Bavaria, Germany). The solution was filtered with a 0.45 μm Acrodisc syringe filter (Pall Corp.; Port Washington, NY, USA) for HPLC analysis. HPLC analysis was performed with a Futecs model NS-4000 HPLC apparatus (Daejeon, Korea) and a C18 column (μBondapak™ C18 10 μm 125Å 3.9 × 300 nm column, Waters, Milford, MA, USA). The mobile phase had a gradient prepared from mixtures of acetonitrile and 0.25% phosphoric acid, and the column temperature was maintained at 30 °C. The flow rate was maintained at 0.7 mL/min. Twenty microliters of the solution was injected into the HPLC, and the detection wavelength was set at 221 nm. The analysis was performed using three biological replicates.

### 4.6. Statistical Analysis

The data for gene expression and valerenic acid and its derivatives contents were analyzed by Microsoft EXCEL (Version. 2010, Microsoft Corporation, Seattle, WA, USA). Statistical analyses for the relative peak areas were conducted using XL-STAT, version 2013 (Addinsoft, NY, USA).

## Figures and Tables

**Table 1:** Composition of volatile compounds in different organs from *V. fauriei*.

No.	Volatile Compounds	RI *	Relative Peak Area (%)			
			Flower	Stem	Leaf	Root
1	*n*-Hexanal	1.43	0	0	0	0.162 ± 0.004
2	Isovaleric acid	11.96	0	0	0	3.338 ± 0.082
3	Camphene	12.91	0	0	0	3.338 ± 0.082
4	β-Pinene	13.64	0	1.244 ± 0.103	0	0.173 ± 0.0041
5	β-Terpinene	14.51	0	0	0	0.558 ± 0.014
6	Myrcene	15.44	0	0	0	0.465 ± 0.011
7	p-Cymene	17.22	0	0	0	0.085 ± 0.002
8	β-Phellandrene	17.79	1.277 ± 0.111	0	0	0.803 ± 0.020
9	γ-Terpinene	19.09	0.132 ± 0.011	0	3.231 ± 0.262	0.052 ± 0.001
10	Terpinolene	21.35	0	0.901 ± 0.074	0	0.041 ± 0.001
11	Ethyl 3-Hydroxymandelate	21.54	0.197 ± 0.017	0.699 ± 0.058	5.331 ± 0.433	0
12	d-Limonene	22.90	0.282 ± 0.024	0	0	0.082 ± 0.002
13	1,3-Bis-(*p*-carbamoylmethylphenoxy)-2-propanol	23.18	0.512 ± 0.044	0	0	0
14	endo-Borneol	23.60	0	0.125 ± 0.010	11.482 ± 0.932	2.841 ± 0.069
15	1-(4-methoxyphenyl)imidazzoline-2-thione	24.22	0.564 ± 0.049	0.211 ± 0.017	0	0
16	2,2-Dimethyl-3-heptanone	24.41	0	0	0.561 ± 0.046	0
17	1,2,4-Trizol-3-amene	24.87	0.179 ± 0.016	0	0	0
18	Borneol	25.11	0	0	0	22.96 ± 0.561
19	Benzoic acid	25.35	0	0.966 ± 0.080	0	1.522 ± 0.037
20	Benzothialzole	25.87	2.725 ± 0.237	0	0	0
21	Eudesmol	25.95	0	0.354 ± 0.029	0	0.077 ± 0.002
22	2-Isopropyl-5-methyl anisole	26.47	0.987 ± 0.086	0.497 ± 0.041	0	0
23	Myrtenyl acetate	26.73	0	0	2.226 ± 0.181	1.729 ± 0.042
24	2-Hexanoylfuran	27.27	0.610 ± 0.053	0	0.556 ± 0.045	0.132 ± 0.003
25	Perillaldehyde	27.57	0.834 ± 0.072	0	0	1.071 ± 0.026
26	9-oxo-(2,6-dimethylpehenyl)amide 9-*H*-Fluorene-4-carboxylic acid	27.94	1.067 ± 0.093	0.241 ± 0.020	21.436 ± 1.741	0.581 ± 0.014
27	Acetic acid	28.31	0.612 ± 0.053	0	0.353 ± 0.029	0.86 ± 0.021
28	α-Gurjunene	29.74	0.640 ± 0.056	0	0	0.133 ± 0.003
29	Bornyl acetate	30.51	0.132 ± 0.011	0	0	19.923 ± 0.487
30	9*H*-Fluorene-4-carboxylic acid	30.77	0.266 ± 0.023	0.306 ± 0.025	0.253 ± 0.021	0.087 ± 0.002
31	α-Elemene	30.88	0	0	0	0.038 ± 0.001
32	1-Trifluoromethyl-4-(2-emthoxylbenzyloxy)-3-nitro-benzene	31.06	0.298 ± 0.026	0.078 ± 0.006	0	0.029 ± 0.001
33	*N*-(Cyclohexanecarbonyl)-l-proline isobutyl ester	31.35	0.133 ± 0.012	0	0	0.022 ± 0.001
34	Bromopropylate	31.97	0.461 ± 0.040	0.367 ± 0.030	0	0.318 ± 0.008
35	Methadone *N*-oxide	32.50	0.286 ± 0.025	0	2.524 ± 0.205	0.509 ± 0.012
36	Quinoline	33.01	0.191 ± 0.017	0.174 ± 0.014	1.510 ± 0.123	1.35 ± 0.033
37	2-Chloro-6-methyl-pyridine	33.40	0.190 ± 0.016	0.226 ± 0.019	0.456 ± 0.037	0.065 ± 0.002
38	Pentadecane	33.51	0.170 ± 0.015	0	0	0.095 ± 0.002
39	4,6-bis(1-Methylethyl)-trans-1,3-dioxane	34.00	0	0.074 ± 0.006	0.751 ± 0.061	0.065 ± 0.002
40	*o*-Choloroaniline	34.12	0	0.955 ± 0.079	1.886 ± 0.153	0
41	α-Caryophyllene alcohol	34.72	0	0	0	1.272 ± 0.031
42	Isolonhifolan-8-ol	34.81	0.8410.073	0	0.424 ± 0.034	0.209 ± 0.005
43	γ-Elemene	34.93	0	0	0	0.357 ± 0.009
44	Caryophyllene	35.22	0.680 ± 0.059	0	0.205 ± 0.017	0.134 ± 0.003
45	5-(Phenylmethyl)-2-thioxo-4-imidazolidinone	35.37	0	2.235 ± 0.0185	0.434 ± 0.035	0
46	α-Acorenol	36.04	0.284 ± 0.025	0	0	4.025 ± 0.098
47	cis-β-Farnesene	36.18	0	0	0	0.041 ± 0.001
48	Humulene	36.28	0	0	0	0.085 ± 0.002
49	Aromadendrene	36.56	0	0	0	0.741 ± 0.018
50	Alloaromandendrene	36.71	1.162 ± 0.101	0	0	0.066 ± 0.002
51	Ylangene	36.92	0	0.299 ± 0.025	0.779 ± 0.063	2.878 ± 0.070
52	1-(1,5-Dimethyl-4-hexenyl)-4-methyl-benzene	37.13	1.181 ± 0.102	0.297 ± 0.025	0	0.065 ± 0.002
53	β-Ionone	37.39	0.554 ± 0.048	0.179 ± 0.015	0.922 ± 0.075	2.333 ± 0.057
54	Pentadecane	37.62	0	0.523 ± 0.043	0.439 ± 0.036	1.786 ± 0.044
55	*N*-(1*H*-1,3-Benzimidazol-2-ylmethyl)-4-methoxy-benzamide	38.31	1.646 ± 0.143	0.047 ± 0.004	0.629 ± 0.051	0.475 ± 0.012
56	Malonic acid	38.50	0	0	1.177 ± 0.096	3.118 ± 0.076
57	β-Bisabolene	38.81	0	0.158 ± 0.013	0.119 ± 0.010	0.54 ± 0.013
58	cis-Sesquisabinene	38.87	2.302 ± 0.200	0.640 ± 0.053	2.530 ± 0.0205	0.527 ± 0.013
59	*N*-Phenyl-(3-methyl-2-oxiranyl) methyl carbamate	39.03	0	0.362 ± 0.030	0	0.287 ± 0.007
60	Bornyl isovalerate	39.20	1.110 ± 0.096	0.143 ± 0.012	0.674 ± 0.055	0.216 ± 0.005
61	exo-3-Methyl-1,7,7-trimethylbicyclo[2,2,1]hept-2-yl butanoate	39.50	0	0.143 ± 0.012	0	2.464 ± 0.060
62	2.6-Dimethylnon-1-en-3-yn-5-yl valeric acid	39.81	0.708 ± 0.061	0	0	1.249 ± 0.031
63	cis-α-Bisabolene	40.33	0.621 ± 0.054	0	0.533 ± 0.043	0.187 ± 0.005
64	trans-Sesquisabinene	40.50	0	0	0.467 ± 0.038	0.213 ± 0.005
65	1-Phenylthio-3-(1-piperidyl)-propan-2ol	40.61	0.894 ± 0.078	0	0	0.134 ± 0.003
66	6-*epi*-Shyobunol	40.79	0	0.139 ± 0.011	0	2.376 ± 0.058
67	Photocitral B	40.97	0.779 ± 0.068	0.356 ± 0.029	2.119 ± 0.0172	0.282 ± 0.007
68	*N*-(4-Methoxyphenyl)-propanamide	41.42	1.247 ± 0.019	0.339 ± 0.028	1.212 ± 0.098	2.537 ± 0.062
69	Menthyl acetate	41.68	0	0.254 ± 0.021	0.901 ± 0.073	0.08 ± 0.002
70	(−)-Isolongifolol methyl ether	42.09	0	0	0	0.207 ± 0.005
71	5,5-Dimethyl-4-(3-methyl-1,3-butadienyl)-1-oxaspiro[2,5]octane	42.20	1.121 ± 0.097	0	0	0.127 ± 0.003
72	3,3-dichlorodihydro-2(3*H*)-furanone	42.38	0	0	0	0.313 ± 0.008
73	*Z*-9-Pentadecenol	42.74	2.454 ± 0.213	1.530 ± 0.126	3.563 ± 0.289	0.544 ± 0.013
74	Acetonylacetone dioxime	42.97	0	0.122 ± 0.010	0	0
75	1-Adamantylmethyl 3-methyl-2-butenoate	43.27	1.593 ± 0.138	1.392 ± 0.115	0.223 ± 0.018	0.221 ± 0.005
76	3-(Methylthio)phenyl isothiocyanate	43.89	0	0.527 ± 0.044	0.921 ± 0.075	0.041 ± 0.001
77	1-Adamantylmethyl octanoic acid	44.20	0.780 ± 0.068	11.040 ± 0.912	0	0.123 ± 0.003
78	2-(Methylthio)-benzothialzole	44.43	1.013 ± 0.088	0.300 ± 0.025	0.280 ± 0.023	0.06 ± 0.001
79	3-Methyl-2(3*H*)-benzothiazolethione	44.74	0.596 ± 0.052	0.439 ± 0.036	0.654 ± 0.053	0.046 ± 0.001
80	Pentanoic acid	45.25	1.072 ± 0.09	0.531 ± 0.044	0.522 ± 0.042	0.165 ± 0.004
81	Terpinyl acetate	45.42	1.550 ± 0.135	3.000 ± 0.248	0	0
82	*p*-Methoxybenzylazidoformate	46.29	0.975 ± 0.085	2.492 ± 0.206	1.051 ± 0.085	0.439 ± 0.011
83	5-Amino-ethyl ester [1,2,4]triazolo[4.3-*a*]pyrimidine-6-carboxylate	46.47	0.611 ± 0.053	0.288 ± 0.024	0	0
84	1,3,-Trimethyl-2-hydroxymethyl-3,3-dimethyl-4(3-methylbut-2-eny)-cyclohexene	46.71	1.044 ± 0.091	0.766 ± 0.063	0	0
85	5-Butyl-6-hexyloctahydro-1H-indene	47.14	0.992 ± 0.086	0.229 ± 0.019	0	0
86	4-Hydroxy-2-hydroxymethyl-6-methylpyrimidine	47.47	0.456 ± 0.040	0	0	0
87	Murolan-3,9(11)-diene-10-peroxy	47.76	0.888 ± 0.077	0.355 ± 0.029	0.482 ± 0.039	0.131 ± 0.003
88	Methanone	48.04	0.414 ± 0.036	0.2750.023	0	0
89	8-Chlorooctyl isobutyl carbonate	48.29	0.522 ± 0.045	0.134 ± 0.011	0	0
90	1,2-Pentanediol	48.72	0.719 ± 0.062	0	0	0.031 ± 0.001
91	Bicyclogermacrene	49.21	1.642 ± 0.143	0	0	0
92	Larixone	49.39	0	0.159 ± 0.013	0	0
93	Ursane-3,16-diol	49.70	0.856 ± 0.074	0.921 ± 0.076	0.640 ± 0.052	0.04 ± 0.001
94	Dodecahydro-3,8,8,11a-tetramethyl-5*H*-3,5a-epoxynaphth[2,1-*c*]oxepin	49.94	1.248 ± 0.108	0.354 ± 0.029	0	0
95	Hexahydro-5-methyl-1-phenyl-1,3,5-triazine-2-thione	50.27	0	1.515 ± 0.125	0.240 ± 0.019	0
96	(2*R*,4*R*)-*p*-Mentha-6,8-diene-2-hydroperoxide	50.71	0.793 ± 0.069	0.271 ± 0.022	0	0
97	4-(Diethoxyphosphiniyl)butanoic acid	51.18	0.259 ± 0.022	0.246 ± 0.020	0	0
98	2,2-Dimethylpropanoic acid	51.74	0.878 ± 0.076	0.076 ± 0.006	0	0
99	Nerolidol isobutyrate	52.17	0.587 ± 0.051	0.253 ± 0.021	0	0
100	Longifolenaldehyde	52.31	0.510 ± 0.044	0	0	0
101	Caryophyllene oxide	52.67	0.359 ± 0.031	17.454 ± 1.442	0	0
102	Carbamic acid	52.89	0.670 ± 0.058	0.939 ± 0.078	0.220 ± 0.018	0.061 ± 0.001
103	Ptenin-6-carboxylic acid	53.22	1.473 ± 0.127	2.224 ± 0.183	0	0.024 ± 0.001
104	2,4,4-Trimethyl-3-hydroxymethyl-5*a*-(3-methyl-but-2-enyl)-cyclohexene	53.48	0.218 ± 0.019	0.324 ± 0.027	0	0
105	2-(2-Dodecen-1yl)succinic acid	53.76	0.801 ± 0.070	0.145 ± 0.012	0	0
106	Ginsenol	54.37	0.294 ± 0.026	0.394 ± 0.033	0	0
107	Costunolide	54.55	0	0.114 ± 0.009	0	0
108	1-Formyl-2,2,6-trimethyl-3-(3-methyl-but-2-enyl)-6-cyclohexene	54.95	3.140 ± 0.273	1.125 ± 0.093	0	0
109	4-*epi*-Cubedol	55.61	0.819 ± 0.071	0.313 ± 0.026	0	0
110	5-(6-Bromodecahydro-2-hydroxy-2,5,5a,8a-tetramethyl-1-naphthalene)-1,2-pentanediol	55.82	0.441 ± 0.038	0	0	0
111	(8S,14)-Cedran-diol	56.00	0.855 ± 0.074	0.914 ± 0.075	0	0
112	Cedrol	56.39	0.379 ± 0.033	0	0	0
113	8-Propoxy cedrane	56.65	0.187 ± 0.016	0.078 ± 0.006	0	0
114	5,6,6-Trimethyl-undeca-3,4-diene-2,10-dione	56.81	0.311 ± 0.027	0.130 ± 0.011	0	0
115	Octahydro [1,2]azaborino[1,2-*a*][1,2]azaborine	57.53	0.535 ± 0.046	0	0	0
116	2.6-Dimethylnon-1-en-3-yn-5-yl valeric acid	58.56	0.292 ± 0.025	0	0	0
117	Terephthalic acid	58.80	0.174 ± 0.015	7.487 ± 0.618	0	0
118	Adamantane	58.98	0.457 ± 0.040	0.944 ± 0.078	0	0
119	4,7-Methano-3,6,8-methenocyclopent[a]indene	59.80	0.105 ± 0.009	0	0	0
120	4-Dimethylamino-2-methyl-1-phenyl-butan-2-ol	60.11	0.316 ± 0.027	0.376 ± 0.031	0	0
121	(±)cis-3,4-Dimethyl-2-phenyltetrahydro-1,4-thiazine	61.09	0.185 ± 0.016	0	0	0
122	2-Isopropyl-6-phenylnicotinonitrile	62.82	0.251 ± 0.022	0	0	0
123	2-Propenoic acid	64.22	0.242 ± 0.021	0.300 ± 0.025	0	0
124	Hexanedioic acid	64.57	0.476 ± 0.041	7.976 ± 0.659	0	0
125	Methadone *N*-oxide	65.01	0.115 ± 0.010	0	0	0
126	Isophthalic acid	67.21	0.591 ± 0.051	1.164 ± 0.096	0	0
127	Diisooctyl phthalate	68.01	0.276 ± 0.024	0.420 ± 0.035	0	0
128	Di-(2-methyoxyethyl) Isophthalate	69.18	0	0.113 ± 0.009	0	0
Total			65.33 ± 5.564	84.057 ± 6.932	74.916 ± 6.073	95.661 ± 2.342

* Retent ion time (min).

**Table 2:** Amounts of valerenic acid and its derivatives in *V.* *fauriei* roots.

Compound	Dry Weight (μg/g)
valerenic acid	69.450 ± 0.263
acetoxyvalerenic acid	32.234 ± 0.961
hydroxyvalerenic acid	n.d. ^1^

Numbers indicate the mean of three replicates ± standard deviation. ^1^ n.d. = not detected.

## Abbreviations

The following abbreviations are used in this manuscript:

AACT	Acetocetyl-CoA thiolase
CAR/PDMS	carboxen/polydimethylsiloxane
CMK	4-(cytidine 5′-diphosphate)-2-*C*-methyl-d-erythritol kinase
DMAPP	dimethylallyl diphosphate
DXP	1-deoxy-d-xylulose 5-phosphate
DXR	DXP reductoisomerase
DXS	DXP synthase
FDS	farndsyl diphosphate synthase
FPP	farnesyl diphosphate
GA-3P	glyceraldehyde-3-phosphae
GC	gas chromatography
GC/MS	gas chromatography-mass spectrometry
GDS	geranyl diphosphate synthase
GGPP	geranyl geranyl diphosphate
GPP	geranyl diphosphate
HDR	(*E*)-4-hydroxy-3-methylbut 2-enyl diphosphate reductase
HDS	(*E*)-4-hydroxy-3-methylbut-2-enyl diphosphate synthase
HMG-CoA	3-hydroxy-3-methylglutaryl-CoA
HMGR	HMG-CoA reductase
HMGS	HMG-CoA synthase
IDI	isopentenyl diphosphate isomerase
IPP	isopentenyl diphosphate
MCT	2-C-methyl-d-erythritol 4-phosphate cytidylyltransferase
MDS	2-C-methyl-d-erythritol 2,4-cyclodiphosphate synthase
MEP	2-C-methyl-d-erythritol 4-phosphate
MK	mevalonate kinase
MVA	mevalonae
MVD	mevalonate diphosphate decarboxylase
qRT-PCR	quantitative Real-time PCR
PMK	phosphomevalonate kinase
TPS	terpene synthase

## Appendix

**Table A1:** Primers sets used in the experiments and their efficiency (%) for qRT-PCR.

Primer	Sequence (5′ to 3′)	Amplication (bp)	Primer Efficiency (%)
VfAACT-F	ATCGGGCATGAAAGCAACCA	129	97.9
VfAACT-R	GATCCCTTCCTTGCTTCCGCTA		
VfHMGS-F	TGGTGGAACTGCAGCATTGTTC	131	98.8
VfHMGS-R	CCGCACTGTCTGTGCACACGA		
VfHMGR-F	TCAGATGCCCTGCCTCTTCC	102	100.2
VfHMGR-R	GATCTTCTCGCGCCACCTTG		
VfMK-F	CGGCGGCTGCGTATTGACT	133	107.1
VfMK-R	GCAAATCTCGAGCCCATTGC		
VfPMK-F	GAGCCGGAATCACAGACGGA	146	93.3
VfPMK-R	CTCCACGTCTTTGCGAGGCT		
VfMVD-F	AATGGAATCGCGCTGAAGGA	105	98
VfMVD-R	CACAGCATTCGGCCCAGCAT		
VfIDI-F	AGCAGATGCAGGCGAAGAGG	111	93.3
VfIDI-R	GCCTCGCTTAAGTTCCCGTTCT		
VfFDS-F	TGATGACGACGGCAAGGAGA	131	94.9
VfFDS-R	CACCAACCAAGTGAGCATGCAAGA		
VfDXS-F	GCCCAATACCACCTGTCGGA	121	95.8
VfDXS-R	TGCATCGGTCCACCAATCTG		
VfDXR-F	AGAACTCCGGTCATTGTGCCA	120	100.1
VfDXR-R	CCGCCTCGATCTTTGCAAGTTA		
VfMCT-F	TCAGTTGCTCTGCAAATGGGAGT	104	102.2
VfMCT-R	TCCCATTCTTGTGCCCTTTCC		
VfCMK-F	GCACCATTGTTGGGATCGGT	113	82.6
VfCMK-R	CTCGTTCTCGGCTCGTGTGA		
VfMDS-F	TGCAGCTACTGCTGCTGTGGA	144	70.6
VfMDS-R	GGTATGTTGATGCCGCCGAT		
VfHDS-F	CTGACAGGCGGGCACAGTTT	134	97.1
VfHDS-R	GCCGATTCGCATAGCTCTTCC		
VfHDR-F	CCGAAGCAATCGGGAAGTTG	112	95
VfHDR-R	CGCTCTTGAGTAGCGTCGCA		
VfGDS-F	TAGCAGTGCTGGCGGGAGAT	145	102.8
VfGDS-R	TCGCGCGTCGTACTCATTTG		
